# Extraction of the Rashba spin-orbit coupling constant from scanning gate microscopy conductance maps for quantum point contacts

**DOI:** 10.1038/s41598-017-14380-2

**Published:** 2017-11-02

**Authors:** K. Kolasiński, H. Sellier, B. Szafran

**Affiliations:** 1 AGH University of Science and Technology, Faculty of Physics and Applied Computer Science, al. Mickiewicza 30, 30-059 Kraków, Poland; 2 Université Grenoble Alpes, CNRS, Institut Néel, 38000 Grenoble, France

## Abstract

We study the possibility for the extraction of the Rashba spin-orbit coupling constant for a two-dimensional electron gas with the conductance microscopy technique. Due to the interplay between the effective magnetic field due to the Rashba spin-orbit coupling and the external magnetic field applied within the plane of confinement, the electron backscattering induced by a charged tip of an atomic force microscope located above the sample leads to the spin precession and spin mixing of the incident and reflected electron waves between the QPC and the tip-induced 2DEG depletion region. This mixing leads to a characteristic angle-dependent beating pattern visible in the conductance maps. We show that the structure of the Fermi level, bearing signatures of the spin-orbit coupling, can be extracted from the Fourier transform of the interference fringes in the conductance maps as a function of the magnetic field direction. We propose a simple analytical model which can be used to fit the experimental data in order to obtain the spin-orbit coupling constant.

## Introduction

Spin-orbit (SO) coupling in semiconductor nanostructures^1^ besides its direct effects in spin relaxation and dephasing^2–4^, induce appearance of a number of extensively studied phenomena, including spin Hall effects^5–7^, persistent spin helix states^8–10^, or Majorana fermions^11^ in contacts with superconductors. Moreover, the SO interactions allow for construction of spin-active elements of spintronic devices, e.g. spin transistors^12–16^, exploiting the precession of the electron spin in the SO effective magnetic field^17^ or spin-filters based on quantum point contacts (QPCs)^15,18^.

In the two-dimensional electron gas (2DEG) formed at the semiconductor heterojunctions a strong Rashba spin-orbit interaction appears as a consequence of electric fields present within the confinement layer. The latter results from the electrostatic potential of the ionized donor layer that provide the charge to the 2DEG, which produces the the Rashba SO coupling^19^. The characterization of the Rashba interaction strength is of an elementary importance for the design of spin devices and description of the SO transport phenomena. Measurements of the SO coupling usually employ the Shubnikov-de Haas^20–27^ oscillations or antilocalization in the magnetotransport^28^. Optical procedures using photocurrents^29^ or precession of optically polarized electron spins^17^ are also employed. The spin-orbit coupling constant for a disordered sample can be extracted from QPC conductance for rotated external magnetic field^30^.

The Rashba SO coupling produces a shift of the spin-up and spin-down dispersion relations along the wave vector axis^1^ that is a linear function of the SO coupling constant. In this paper we propose a way to extract the structure of the dispersion relation near the Fermi level^1^ using spin-dependent scattering and the resulting interference with the scanning gate microscopy^31,32^ (SGM) applied to systems with QPCs^33,34^. In this technique, the tip acts as a floating perturbation of the potential landscape as seen by the Fermi level electrons. As a result the recorded SGM images contain interference fringes due to the incident and backscattered electron waves^35,36^. In presence of an in-plane magnetic field the fringes form beating pattern due to spin-dependence of the Fermi wavelengths^37^. In this paper we analyze the beating patterns that appear for SO-coupled high-mobility systems. The electron – when scattered – experiences precession of its spin due to rotation of the momentum-dependent effective magnetic field^17^, and the interference of the incident and reflected electron waves potentially involves spin-mixing effects. However, we find that in the absence of the external magnetic field the backscattering involves a pure inversion of the effective field with no precession effects. The latter are triggered by an external in-plane magnetic field, and lead to an appearance of the dependence of the beating patterns on the orientation of the magnetic field. We demonstrate that the shape of the Fermi level structure and thus the SO coupling constant can be traced back from the beating patterns by the Fourier transform analysis.

The dispersion relation including the spin-orbit coupling effects for the 2DEG confined at the metal surface^38–44^, or graphene^45^ can be experimentally determined by well established techniques of the scanning tunneling spectroscopy (STS)^38,39,45^, or angle-resolved photoemission spectroscopy (ARPES)^40–44^. The advantage of using the SGM technique over STS and ARPES is that SGM exploits the long-range perturbation of the electrostatic potential introduced by the charged probe and does not require the electrons from 2DEG to leave the surface of the system. For that reason SGM is not limited to the surface 2DEG and can be used for the electron gas burried at a distance from the surface, e.g. within the semiconductor heterostructure^31,33–37^, at a depth of about 50 nm.

## Theory

The theoretical approach^46,47^ applied in this paper has been recently verified in interpretation of the experimental SGM maps for QPCs in both disordered and high-mobility samples^46^. Below we present a proposal of a procedure for extraction of the Rashba constant using rotation of the external magnetic field within the plane of 2DEG confinement followed by a Fourier transform post-treatment.

**Figure 1:**
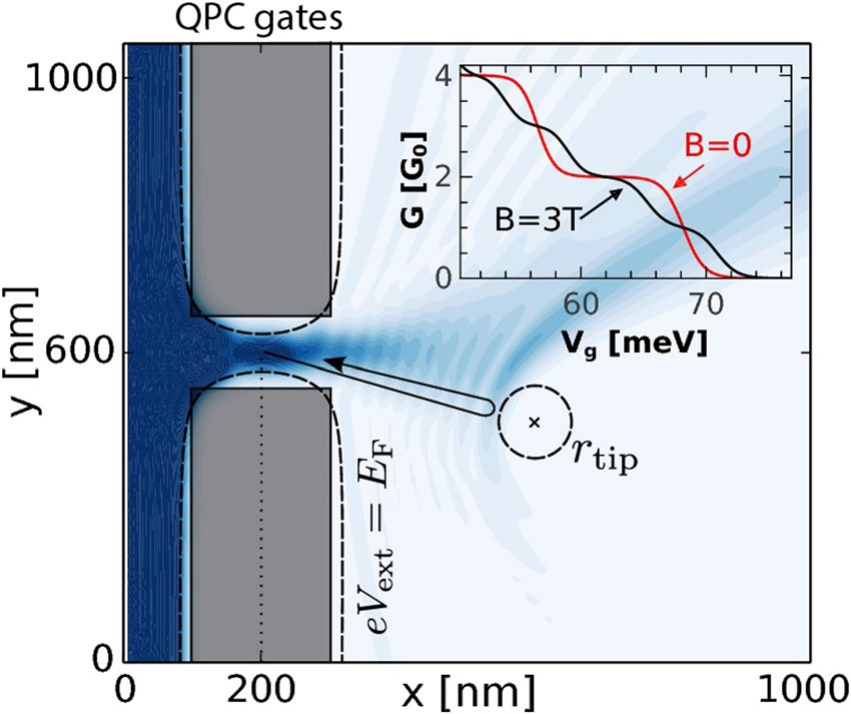
Sketch of system. The electrons come from the reservoir on the left of the QPC. The computational box starts at *x* = 0 nm. The QPC gates form a gap of size 200 nm × 100 nm centered at (200 nm, 600 nm). The gates are located at 50 nm above the 2DEG layer^51^. Dashed lines show the potential energy isolines for which *eV*_ext_ = *E*_*F*_ in leads. The SGM tip is located at ${r}_{{\rm{tip}}}=({x}_{{\rm{tip}}},{y}_{{\rm{tip}}},50\,{\rm{nm}})\mathrm{.}$ The blue map shows (the square root of) the scattering electron density obtained for the QPC tuned to the first conductance plateau [*G* = 2*G*_0_ with ${G}_{{\rm{0}}}=\frac{{e}^{2}}{h}$] for electrons incident from the left lead for *B* = 0. The inset presents the standard conductance quantization as a function of gate voltage *V*_g_ in case without and with in-plane magnetic field.

We consider Fermi level transport in a 2DEG within In_0.5_Ga_0.5_As with a local constriction forming the QPC as depicted in Fig. 1. The Fermi level electrons travel from the electron reservoir placed at *x *< 100 nm through a channel modeled with an infinite potential step and an additional potential tuned by gates (gray areas of the scheme). A negatively charged tip acts as a backscatterer to the right of the QPC. The conductance maps as functions of the tip position resolve the coherent interference fringes as observed in a number of experiments^48,33,35,36,49^. The part of the system to the right of the QPC is considered open such that electron may freely propagate without reflections. Transparent boundary conditions for the electron flow are introduced with a method described in ref.^50^.

We adopt a standard two-dimensional model assuming that all the electrons of 2DEG occupy a strongly localized lowest-energy state of the vertical quantization. The Hamiltonian accounts for the Rashba SO interaction and a presence of the external magnetic field applied within the plane of confinement

1 $$H=[\frac{{\hslash }^{2}}{2{m}_{{\rm{eff}}}}{{\boldsymbol{k}}}^{2}+e{V}_{{\rm{ext}}}(r)]{\boldsymbol{I}}+\frac{1}{2}g{\mu }_{{\rm{B}}}{\boldsymbol{B}}\cdot {\boldsymbol{\sigma }}+{{\boldsymbol{H}}}_{{\rm{rsb}}}$$

with $k=-i\nabla -e{\boldsymbol{A}}$, ${\boldsymbol{B}}=({B}_{{\rm{x}}},{B}_{{\rm{y}}}\mathrm{,0})$, and ***σ*** is the vector of Pauli matrices. The external potential *V*_ext_ is a superposition of two components: *(i) V*_QPC_ – the QPC gate potential modeled with analytical formulas for a rectangle gate adapted from ref.^51^, and *(ii) V*_tip_ – the electrostatic potential created by the charged tip of the scanning probe. The tip potential is modeled by the Lorentzian profile given by ${V}_{{\rm{tip}}}={d}_{{\rm{tip}}}^{2}{V}_{{\rm{t}}}/[{(x-{x}_{{\rm{tip}}})}^{2}+{(y-{y}_{{\rm{tip}}})}^{2}+{d}_{{\rm{tip}}}^{2}],$ with effective width *d*_tip_ = 50 nm, which is of order of the distance between 2DEG and surface of the sample, and *V*_t_ that depends on the voltage applied to the tip. This form of the potential results from the screening of the tip charge by 2DEG^52,53^. The Rashba Hamiltonian ${{\boldsymbol{H}}}_{{\rm{rsb}}}=\gamma \{{{\boldsymbol{\sigma }}}_{x}{k}_{y}-{{\boldsymbol{\sigma }}}_{y}{k}_{x}\}$ in Eq. (1) comes from the electrostatic confinement of the 2DEG in the growth direction^54^. We apply the symmetric gauge ${\boldsymbol{A}}=({B}_{y}z,{B}_{x}z,\mathrm{0)}$. By choosing the plane of the 2DEG confinement to be located at *z *= 0, we get ***A*** **= 0**, thus the magnetic field enters the Hamiltonian only via the spin Zeeman term.

## Results and Discussion

**Figure 2:**
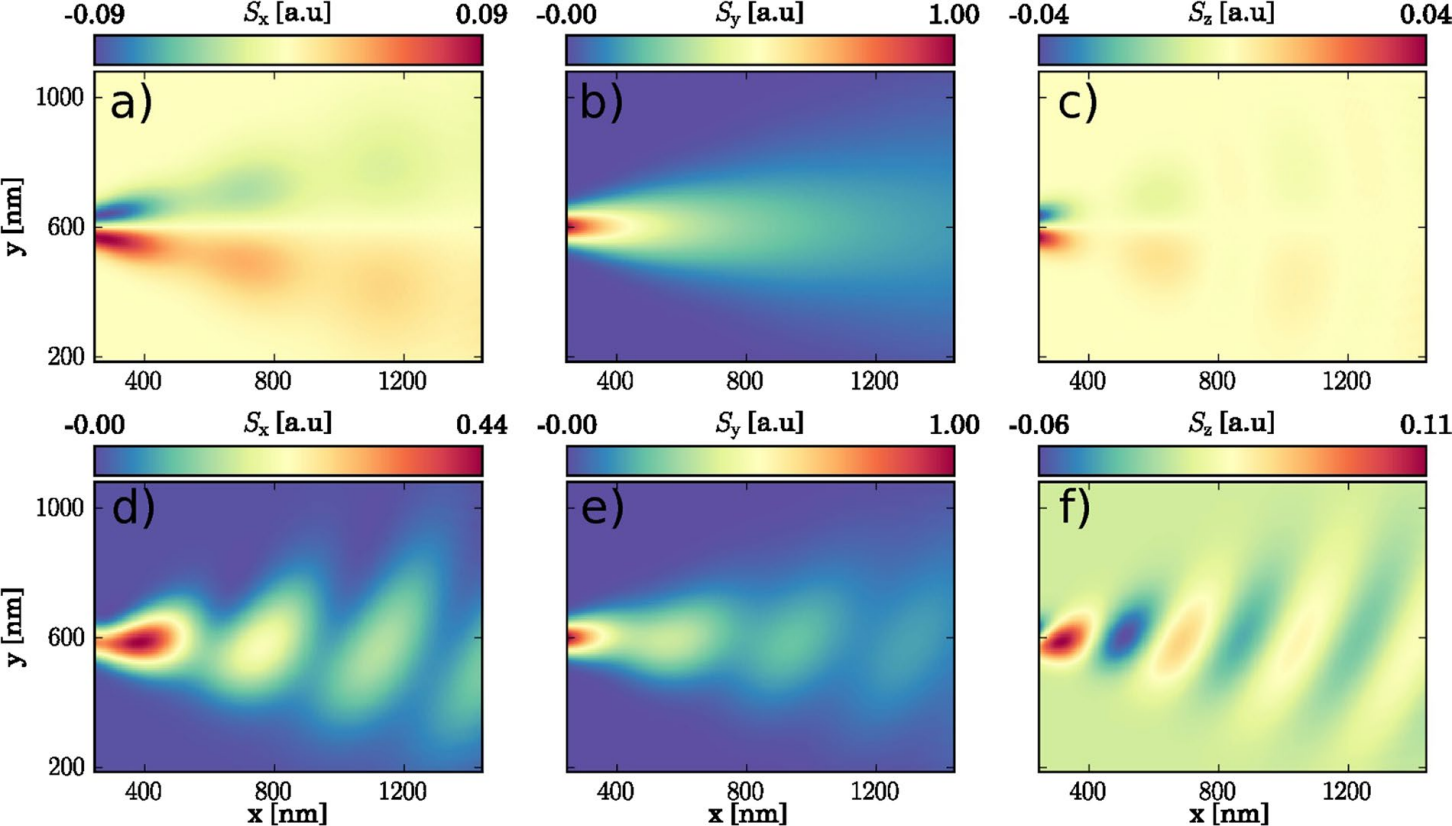
The spin density in the absence of the tip for the electron injected from the *S*_*y*_ > 0 mode of the QPC at *B* = 0. Panels (**a**–**c**) show the spin components for *B* = 0 and *B*_*x*_ = 4 T (**d**–**f**). The densities are given in arbitrary units.

Figure 2 shows the spin density for electrons incident to the system from the positive *S*_*y*_ mode of the QPC, i.e. for electrons with a spin initially set to ${S}_{y}=\hslash \mathrm{/2}$. In presence of SO interaction, this initial spin state is not conserved, but instead evolves during the electron propagation by precessing around the total magnetic field, which is the sum of the local SO field and the uniform external field. In absence of external magnetic field [Fig. 2(a–c)], the local SO field is almost aligned with the initial spin along the *y*-axis, and the initial spin is then almost conserved during the electron propagation. For wave vectors which are off the axis of the system however, the local SO field is not exactly aligned with the initial spin, and the resulting spin precession causes the appearance of small *S*_*x*_ and *S*_*z*_ spin components. In presence of an external magnetic field oriented along the *x*-axis [Fig. 2(d–f)], the direction of the total field is far from the direction of the initial spin state, and the resulting spin precession produces large *S*_*x*_ and *S*_*z*_ spin components which are interlaced with the *S*_*y*_ component. Note that the total field is drastically changed when the electron leaves the lateral confinement of the QPC due to the increase of the wave vector. The direction of the total magnetic field is then different within the constriction and outside. For a system containing only a QPC, the spin precession has no effect on conductance, since the electrons keep moving away from the constriction. When the SGM tip is added to the system however, the spin precession superposes on the backscattering ripples. In the following, we explain how the SGM measurement allows for extraction of the spin modulation for the backscattered electron wave.

**Figure 3:**
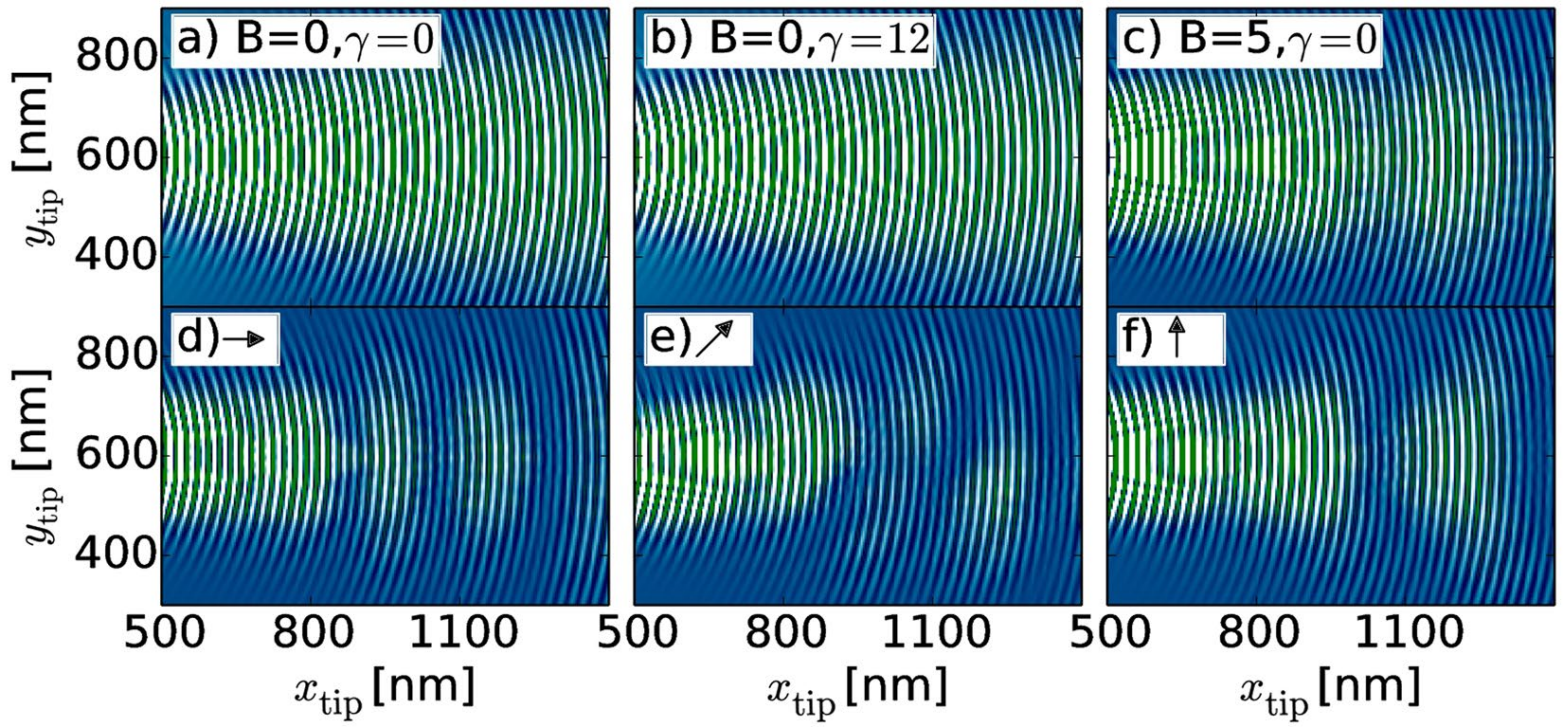
Derivatives of simulated SGM images ($dG/d{x}_{{\rm{tip}}}$) obtained for QPC tuned to the first plateau in arb. units. $dG/d{x}_{{\rm{tip}}}$ map obtained in absence of external magnetic field and SO interaction (**a**), with SO coupling (*γ* = 12 meVnm) at *B* = 0 (**b**), for in-plane magnetic field *B* = 5 T and without SO interaction (**c**). (**d–f**) $dG/d{x}_{{\rm{tip}}}$ images obtained for in-plane magnetic field *B* = 5 T and *γ* = 12 meVnm. The arrows show the in-plane direction of the *B* vector.

Figure 3(a–f) show spatial derivatives of SGM images $dG/d{x}_{{\rm{tip}}}$ obtained from the solution of the quantum scattering problem for QPC depicted in Fig. 1 tuned to the first QPC conductance plateau. For *B* = 0, *γ *= 0 [Fig. 3(a)] a pronounced interference pattern of the incident and backscattered wave is observed^33–36^, with the period of ${\lambda }_{F}\mathrm{/2}$ for both *γ *= 0 [Fig. 3(a)] and $\gamma \ne 0$ [Fig. 3(b)]. A beating pattern^37^ appears at non-zero *B* [Fig. 3(c)], which depends on the orientation of the in-plane field for $\gamma \ne 0$ (Fig. 3(d–f)).

The beating pattern observed in Fig. 3(d) qualitatively reproduces the spin precession shown in Fig. 2(f) for a magnetic field applied along the *x*-axis. Both phenomena have indeed the same origin: the precession effect is the dynamical evolution of a spin in the total effective field, and the beating pattern in the SGM map results from the mixing of the eigenmodes calculated for the same total effective field.

**Figure 4:**
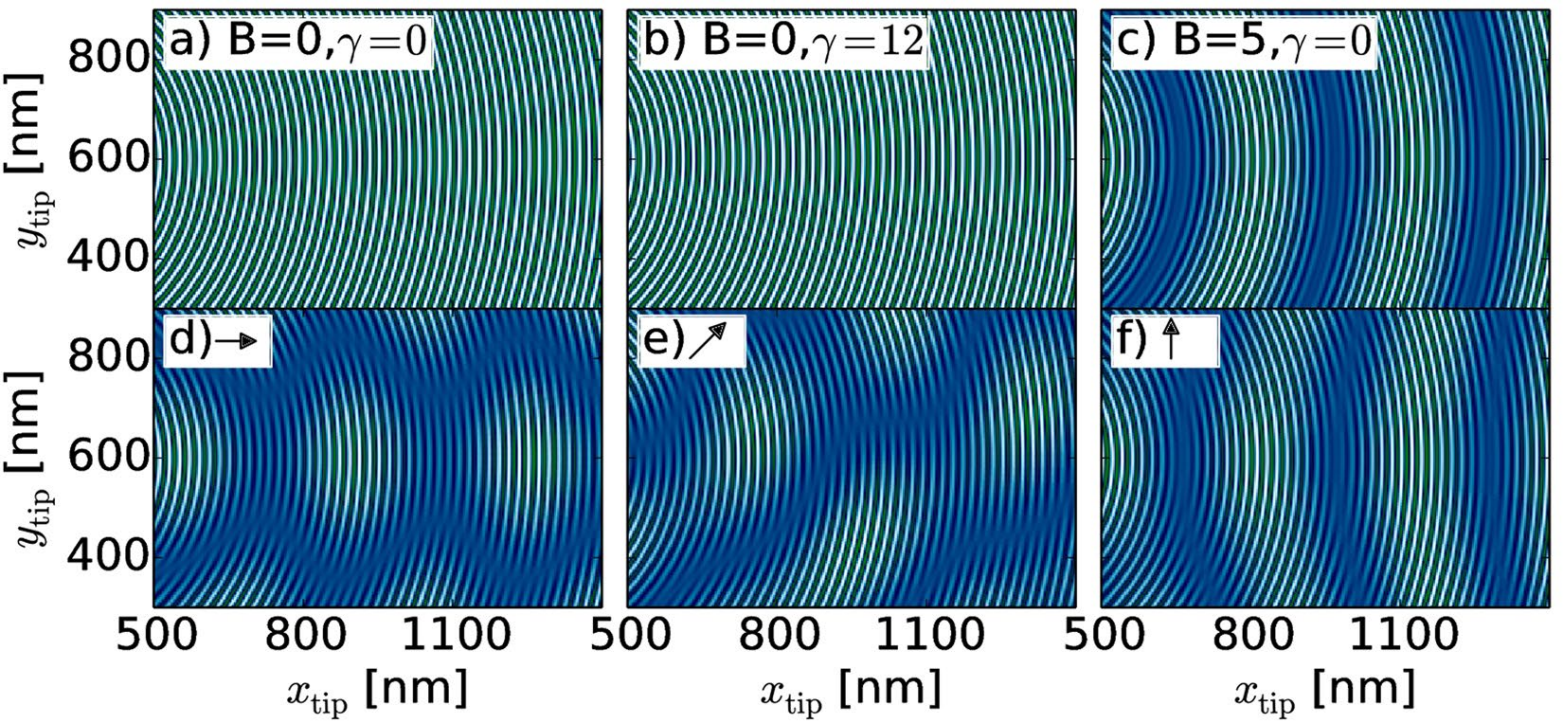
Same as on Fig. 3 but calculated from simple model discussed in this paper.

**Figure 5:**
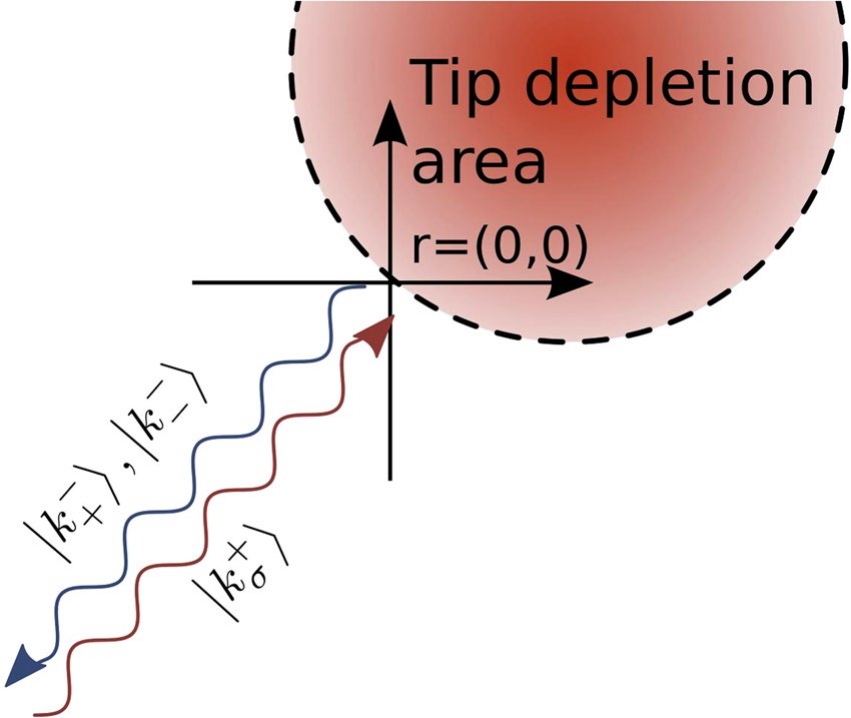
Sketch of considered scattering process. The electron wave leaves QPC in one of two spin states, propagates to the right and is backscattered at position *r* = (0, 0) by the potential barrier induced by the SGM tip. Here we assume a hard wall potential profile (i.e. ${V}_{{\rm{tip}}}=+\infty$ inside the circle).

In order to explain quantitatively the results of Fig. 3 we consider a simple model for SGM images in presence of in-plane magnetic field and SO interaction. The electron wave which leaves the QPC^55–58^ is approximated by a plane wave *e* ^*ikr*^ (an inverse of the square root of the distance from the QPC is neglected as slowly varying). The schematics of the considered scattering process is presented in Fig. 5. The electron wave which leaves the QPC (not shown in the diagram) propagates through the device until it is backscattered by the potential barrier created by the SGM tip with probability 1. We fix the origin at the scattering point. For a given incoming spin state $|{k}_{\sigma }^{+}\rangle$ the scattering wave function can be expanded in terms of the possible scattering modes

2 $$|{{\rm{\Psi }}}_{\sigma }\rangle ={e}^{i{k}_{\sigma }^{+}r}|{k}_{\sigma }^{+}\rangle +{{\rm{\Sigma }}}_{\sigma ^{\prime} }{a}_{\sigma \sigma ^{\prime} }{e}^{-i{k}_{\sigma ^{\prime} }^{-}r}|{k}_{\sigma ^{\prime} }^{-}\rangle ,$$

where ${k}_{\sigma }^{\pm }r=|{{\boldsymbol{k}}}_{\sigma }^{\pm }\cdot {\boldsymbol{r}}|$ and ${k}_{\sigma }^{\pm }$ denotes the absolute value of the wave vector of an electron in spin state *σ*. The sign in the superscript indicates the electron incoming from left+ or backscattered by the tip −. The values of the scattering amplitudes ${a}_{\sigma \sigma ^{\prime} }$ depend on a specific situation. For SO coupling and magnetic field simultaneously present, the Hamiltonian for a free electron can be written

3 $${\boldsymbol{H}}=[\begin{array}{cc}{{\boldsymbol{E}}}_{{\rm{kin}}} & \gamma ({{\boldsymbol{k}}}_{{\rm{y}}}+i{{\boldsymbol{k}}}_{{\rm{x}}})+{\alpha }_{{\rm{x}}}-i{\alpha }_{{\rm{y}}}\\ \gamma ({{\boldsymbol{k}}}_{{\rm{y}}}-i{{\boldsymbol{k}}}_{{\rm{x}}})+{\alpha }_{{\rm{x}}}+i{\alpha }_{{\rm{y}}} & {{\boldsymbol{E}}}_{{\rm{kin}}}\end{array}],$$

where ${\alpha }_{x/y}=\frac{1}{2}g{\mu }_{B}{B}_{x/y}$ and ${{\boldsymbol{E}}}_{{\rm{kin}}}=\frac{{\hslash }^{2}{{\boldsymbol{k}}}^{2}}{2{m}_{{\rm{eff}}}}$. Plane wave solution for the Schröedinger equation gives two eigenvalues

4 $${E}_{\sigma }=\frac{{\hslash }^{2}{{\boldsymbol{k}}}^{2}}{2{m}_{{\rm{eff}}}}+\sigma |{\boldsymbol{p}}|,$$

where ${{\boldsymbol{p}}}^{\pm }=(\gamma {k}_{{\rm{y}}}^{\pm }+{\alpha }_{{\rm{x}}},-\gamma {k}_{{\rm{x}}}^{\pm }+{\alpha }_{{\rm{y}}})$, with $\sigma =\{+,-\}$ and

5 $$|{k}_{\sigma }^{\pm }\rangle =\frac{1}{\sqrt{2}}(\begin{array}{c}1\\ \sigma \frac{{p}_{{\rm{x}}}^{\pm }+i{p}_{{\rm{y}}}^{\pm }}{{p}^{\pm }}\end{array}),$$

are eigenvectors for incoming +  and outgoing directions − of an electron, with ${p}^{\pm }$ being the length of ${{\boldsymbol{p}}}^{\pm }$ vector. Due to the assumed infinite potential generated by the SGM tip, the scattering wave function in Eq. (2) has to vanish at *r* = 0 (see Fig. 5)

$${{\rm{\Psi }}}_{\sigma }(r=0)=|{k}_{\sigma }^{+}\rangle +{{\rm{\Sigma }}}_{\sigma ^{\prime} }{a}_{\sigma \sigma ^{\prime} }|{k}_{\sigma ^{\prime} }^{-}\rangle =0.$$

By substituting Eq. (5) to this equation one evaluates the scattering amplitudes ${a}_{\sigma \sigma ^{\prime} }$.

For the special case when SO interaction and magnetic field are not present in the Hamiltonian (3) the propagating modes in Eq. (5) reduce to

$$|{k}_{+}^{\pm }\rangle =|{k}_{+}\rangle =(\begin{array}{c}1\\ 0\end{array}),\,\,|{k}_{-}^{\pm }\rangle =|{k}_{-}\rangle =(\begin{array}{c}0\\ 1\end{array}),$$

with ${k}_{\sigma }^{\pm }=k$ and scattering amplitudes ${a}_{\sigma \sigma ^{\prime} }=-{\delta }_{\sigma \sigma ^{\prime} }$, hence backscattering does not change the spin orientation. The scattering wave function from Eq. (2) is then

$$|{{\rm{\Psi }}}_{\sigma }\rangle ={e}^{ikr}|{k}_{\sigma }^{+}\rangle -{e}^{-ikr}|{k}_{\sigma }^{-}\rangle =({e}^{ikr}-{e}^{-ikr})|{k}_{\sigma }\rangle ,$$

and the scattering density is given by ${\rho }_{\sigma }=\langle {{\rm{\Psi }}}_{\sigma }|{{\rm{\Psi }}}_{\sigma }\rangle \propto cos(2kr),$ and the variation of the *G* map follows the pattern of the density^55^. The SGM conductance pattern can be approximated by $G({r}_{\mathrm{tip}})\,\propto {\cos }({2{kr}}_{\mathrm{tip}})$. The SGM image obtained with this model is presented in Fig. 4(a) and is consistent with the simulated image obtained in Fig. 3(a).

For *B* = 0 and $\gamma \ne 0$ one may easily check that the propagating modes [Eq. (5)] still satisfy orthogonality relations $\langle {k}_{\sigma ^{\prime} }^{+}|{k}_{\sigma }^{-}\rangle ={\delta }_{\sigma \sigma ^{\prime} }$ and $|{k}_{\sigma }^{+}\rangle =|{k}_{\sigma }^{-}\rangle$, which leads to the spin conserving reflection ${a}_{\sigma \sigma ^{\prime} }=-{\delta }_{\sigma \sigma ^{\prime} }$. However, in this case ${k}_{\sigma }^{+}\ne {k}_{\sigma }^{-}$ and the scattering wave function is given by $|{{\rm{\Psi }}}_{\sigma }\rangle =({e}^{i{k}_{\sigma }^{+}r}-{e}^{-i{k}_{\sigma }^{-}r})|{k}_{\sigma }^{+}\rangle$. The electron density is then proportional to ${\rho }_{\sigma }\propto cos([{k}_{\sigma }^{+}+{k}_{\sigma }^{-}]r)$. Since ${k}_{\sigma }^{\pm }={k}^{\pm }+\sigma \frac{\gamma {m}_{{\rm{eff}}}}{{\hslash }^{2}}$ ^59^ we get the same expression as for *γ* = 0 i.e. ${\rho }_{\sigma }\propto \,\cos (2kr)$, which does not depend on electron spin. Hence the SO effect vanishes for the backscattering process which leads to the same SGM image [Fig. 4(b)] as in case of *γ* = 0 [Fig. 4(a)].

The third possible configuration of parameters i.e. *γ* = 0 and $B\ne 0$ was recently discussed in ref.^37^. In this case the same orthogonality relation is still satisfied $\langle {k}_{\sigma ^{\prime} }^{+}|{k}_{\sigma }^{-}\rangle ={\delta }_{\sigma \sigma ^{\prime} }$, and ${a}_{\sigma \sigma ^{\prime} }=-{\delta }_{\sigma \sigma ^{\prime} }$. However, the resulting electron density is now proportional to ${\rho }_{\sigma }\propto \,\cos (2{k}_{\sigma }r)$ and depends on the spin via the Zeeman term inducing shifts of ${k}_{\sigma }$. The approximated SGM map $G={G}_{{\rm{0}}}{\sum }_{\sigma }{T}_{\sigma }\,\cos \,(2{k}_{\sigma }r)$ gives a signal being a superposition of two frequencies ${\omega }_{\sigma }=2{k}_{\sigma }$ resulting in the beating pattern visible in Fig. 4(c). The present reasoning explains the findings of ref.^37^.

In a general case of $B\ne 0$ and $\gamma \ne 0$ the eigenvalues [Eq.(4)] depend on both the direction of the magnetic field and the propagation vector, thus the spin will not be conserved anymore during the backscattering process, since the orthogonality relations between the incident and backscattered modes no longer hold $\langle {k}_{{\sigma }^{^{\prime} }}^{+}|{k}_{\sigma }^{-}\rangle \ne {\delta }_{\sigma {\sigma }^{^{\prime} }}$, and ${a}_{\sigma {\sigma }^{^{\prime} }}\ne -{\delta }_{\sigma {\sigma }^{^{\prime} }}$. The resulting electron density will be then a composition of four different possible superpositions of the Fermi wave vectors ${k}_{i}=\{{k}_{+}^{+}+{k}_{+}^{-},{k}_{+}^{+}+{k}_{-}^{-},{k}_{-}^{+}+{k}_{+}^{-},{k}_{-}^{+}+{k}_{-}^{-}\}$. The SGM images obtained for this general case for three different orientation of magnetic field $\alpha =\{{0}^{\circ },\,{45}^{\circ },\,{90}^{\circ }\}$ are depicted in Fig. 4(d–f). Although, the images differ somewhat from Fig. 3 (d–f), still both the model and the full simulation allow for extraction of the wave vectors and their dependence on the orientation of the magnetic field in the Fourier analysis (see below).

**Figure 6:**
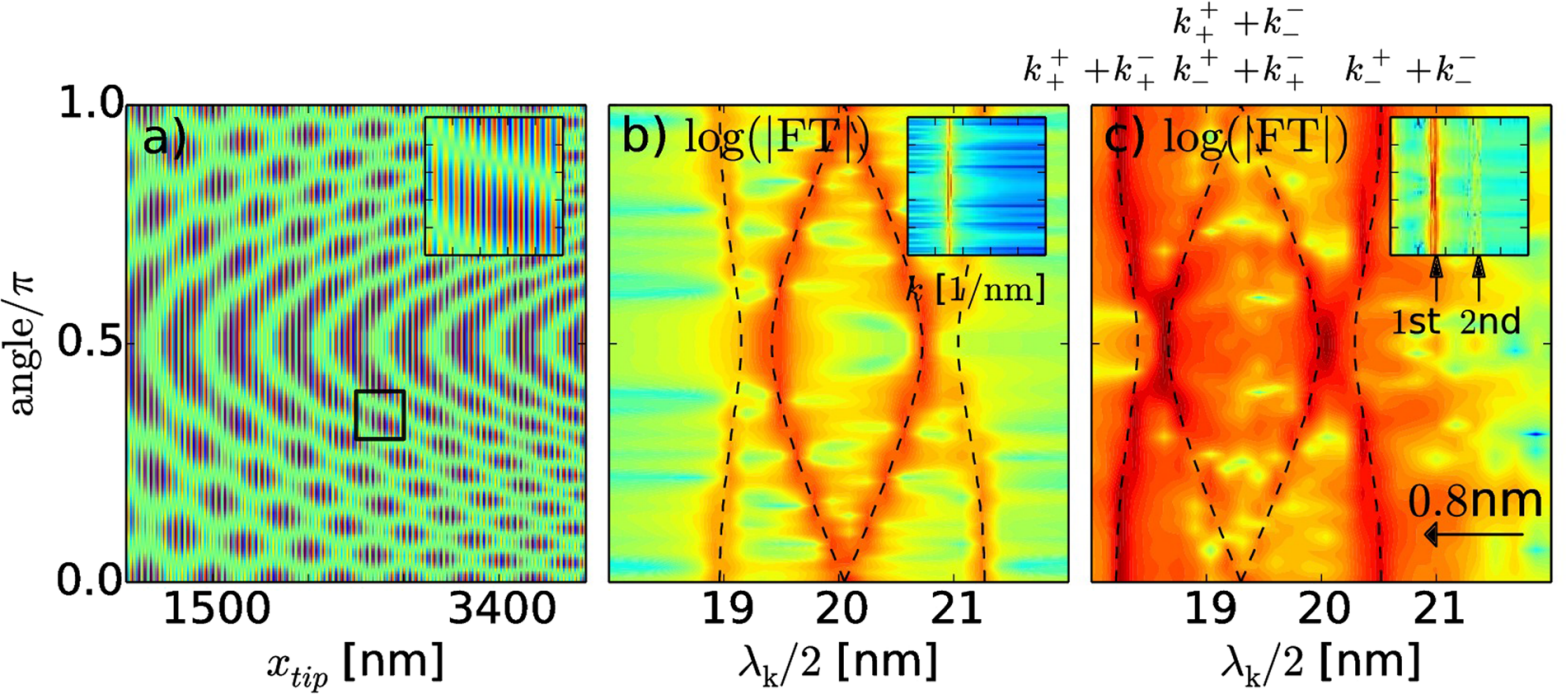
(**a**) Conductance as a function of the tip position moving along the *x* axis (with *y*_tip_ = 600 nm) and the angle that the magnetic field vector forms with the *x* axis. The inset shows a zoomed part in the area denoted by the black square. The simulation was performed for 5 T and *γ* = 12 meVnm with the simple analytical model. (**b**) Fourier transform (FT) of (**a)** remapped from *k* space to $\lambda =2\pi /k$. Dashed lines were calculated from the dispersion relation defined by Eq. (4) as ${\lambda }_{i}=2\pi /{k}_{i}$ with ${k}_{i}=\{{k}_{+}^{+}+{k}_{+}^{-},{k}_{+}^{+}+{k}_{-}^{-},{k}_{-}^{+}+{k}_{+}^{-},{k}_{-}^{+}+{k}_{-}^{-}\}$. The inset shows the same image but in the *k* space for a large range of wave vectors values. (**c**) Same as (**b**) but for the full numerical simulation taken at *G* = *G*_0_. The finite size of the SGM tip potential leads to a shift of all lines towards higher frequencies. Quantum mechanical simulation reveals also the higher harmonics in the inset denoted by 1st and 2nd arrows.

The form of Eq. (3) indicates that rotation of a SGM tip position along the arc centered at the QPC entrance is equivalent to a rotation of the in-plane magnetic field (in an opposite direction) for a fixed tip position. For a practical implementation of an experiment it should be more efficient to perform a SGM scan along a straight line, where the longest electron branch^48,49^ is present and rotate the magnetic field instead (see Fig. 6(a)).

**Figure 7:**
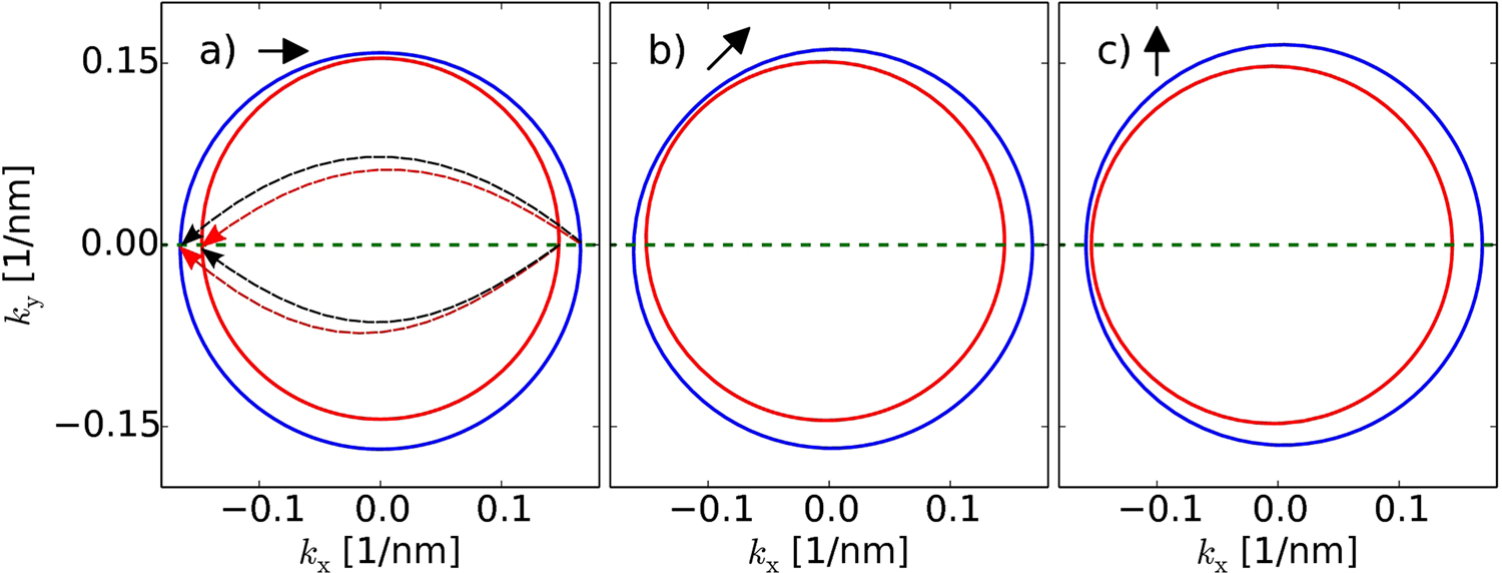
(**a**–**c**) Fermi level surface calculated from Eq. (4) obtained for three directions of magnetic field *ϕ* = {0°, 45°, 90°} (for *B* = 5*T*) denoted by arrows. Green dashed lines show the direction of scattering process i.e. ***k*** = (*k*_x_, 0). Dashed arrows on (**a**) represent four different possible backscattering processes. However, due to the symmetry of the scattering process two of them lead to the same frequency in FT image, hence three lines are observed in Fig. 6(b,c) for $\varphi =0$ and *π*. This is no more valid for (**b**) and (**c**), which imply four different lines in Fig. 6 for other values of *ϕ*.

In Fig. 6(b,c) we present the Fourier transform (FT) of the conductance signal calculated from the $dG/d{x}_{{\rm{tip}}}$ map for the tip moving along the QPC axis, as a function of the magnetic field direction **B** for *B *= 5 T. The results are plotted on the wavelength scale calculated as $\lambda =2\pi /k$. The dashed lines in Fig. 6(b,c) were plotted for backscattering processes that are explained in Fig. 7(a) and calculated numerically from the condition ${E}_{F}={E}_{\sigma }$ with the latter given by Eq. (4). Note, that due to the smooth and extended shape of the tip potential in the full simulation the resonance lines in Fig. 6(c) are slightly shifted to the left by 0.8 nm (in comparison to model Fig. 6(b)). We accordingly shifted the dashed lines in Fig. 6(c) to coincide with the FT image. In the inset in Fig. 6(c) one observes also higher harmonics, which result from the possible multiple reflections between the tip and QPC (not present in the model, see inset in Fig. 6(b)).

The backscattering taken along the axis of the QPC involves *k*_*y*_ = 0 and we find in general four various values of *k*_*i*_ visible as four lines in FT images. However, when *B*_*y*_ = 0, Eq. (4) reduces to

6 $${E}_{\sigma }=\frac{{\hslash }^{2}{k}_{x}^{2}}{2m}+\sigma \sqrt{{\alpha }_{{\rm{x}}}^{2}+{(\gamma {k}_{x})}^{2}},$$

which is symmetric with respect to electron reflection ${E}_{\sigma }({k}_{{\rm{x}}})={E}_{\sigma }(-{k}_{{\rm{x}}})$, which implies the symmetry of scattering process that ${k}_{\sigma }^{+}={k}_{\sigma }^{-}\equiv {k}_{\sigma }$ (see Fig. 7(a)), and thus reducing the number of resonance lines in FT image to three. For other cases presented in Fig. 7(b,c) this symmetry is not satisfied and all four frequencies are visible.

One could expect that there should be another symmetry point when magnetic field is oriented along the SO effective magnetic field i.e. for *α* = 90°. However, for this case *α*_x_ = *k*_*y*_ = 0 thus we get ${E}_{\sigma }=\frac{{\hslash }^{2}{k}_{{\rm{x}}}^{2}}{2m}+\sigma |{\alpha }_{{\rm{y}}}-\gamma {k}_{{\rm{x}}}|$ which in consequence leads to four different spinors $|{k}_{\sigma }^{\pm }\rangle$ (5) and ${a}_{\sigma \sigma ^{\prime} }\ne -{\delta }_{\sigma \sigma ^{\prime} }$. Four different scattering processes leading to four different frequencies are then allowed which thus produces the four resonances observed in FFT images.

Note, that in our previous work^30^ we indicated that for disordered sample with multiple random scatterers the spin-orbit coupling can be estimated by the QPC conductance in the rotated magnetic field. The present paper indicates a solution that is suitable for clean high-mobility two-dimensional electron gas, where the scanning probe experiment induces the backscattering. The beating of conductance due to the spin-orbit coupling modification of the dispersion relation and its modification by the in-plane magnetic field are used for determination of the spin-orbit coupling constant.

## Summary

In summary, we have shown that SGM imaging can be used to extract the Fermi surface properties by Fourier analysis of the beatings due to the SO interaction and an in-plane magnetic field. The analysis allows for deduction of the Rashba constant from the real space measurement of conductance as a function of the tip position involving spin-scattering in a crossed external and built-in magnetic fields.

## Methods

The scattering problem is solved within the finite difference approach^46,47^, with spatial discretization ${\rm{\Delta }}x={\rm{\Delta }}y=6$ nm using the wave function matching (WFM) method^60^. Then we calculate conductance $G$ using the Landauer approach by evaluating $G={G}_{{\rm{0}}}{\sum }_{\sigma }{T}_{\sigma }$ at the Fermi level (with ${G}_{{\rm{0}}}=\frac{{e}^{2}}{h}$). For simplicity, we consider the case of a single mode being transmitted through the QPC ($G\le 2{G}_{{\rm{0}}}$) (see the inset to Fig. 1). We set *E*_*F*_ = 20 meV (for *γ* = 0 the Fermi wavelength is ${\lambda }_{{\rm{F}}}=40$ nm), and the tip potential *V*_t_ = 40 meV for which a strict depletion of the electron density below the tip is obtained (see the dashed circle in Fig. 1). Landé factor is assumed to be *g* = 9 and effective mass *m*_eff_ = 0.0465 *m*_0_ as for InGaAs.

### Data availability

All data generated or analysed during this study are included in this published article.
